# Substantial Agreement of Referee Recommendations at a General Medical Journal – A Peer Review Evaluation at Deutsches Ärzteblatt International

**DOI:** 10.1371/journal.pone.0061401

**Published:** 2013-05-02

**Authors:** Christopher Baethge, Jeremy Franklin, Stephan Mertens

**Affiliations:** 1 Deutsches Ärzteblatt International, Editorial Offices, Cologne, Germany; 2 Department of Psychiatry and Psychotherapy, University of Cologne Medical School, Cologne, Germany; 3 Institute of Medical Statistics, Informatics, and Epidemiology, University of Cologne Medical School, Cologne, Germany; The University of Edinburgh, United Kingdom

## Abstract

**Background:**

Peer review is the mainstay of editorial decision making for medical journals. There is a dearth of evaluations of journal peer review with regard to reliability and validity, particularly in the light of the wide variety of medical journals. Studies carried out so far indicate low agreement among reviewers. We present an analysis of the peer review process at a general medical journal, Deutsches Ärzteblatt International.

**Methodology/Principal Findings:**

554 reviewer recommendations on 206 manuscripts submitted between 7/2008 and 12/2009 were analyzed: 7% recommended acceptance, 74% revision and 19% rejection. Concerning acceptance (with or without revision) versus rejection, there was a substantial agreement among reviewers (74.3% of pairs of recommendations) that was not reflected by Fleiss' or Cohen's kappa (<0.2). The agreement rate amounted to 84% for acceptance, but was only 31% for rejection. An alternative kappa-statistic, however, Gwet's kappa (AC1), indicated substantial agreement (0.63). Concordance between reviewer recommendation and editorial decision was almost perfect when reviewer recommendations were unanimous. The correlation of reviewer recommendations and citations as counted by Web of Science was low (partial correlation adjusted for year of publication: −0.03, n.s.).

**Conclusions/Significance:**

Although our figures are similar to those reported in the literature our conclusion differs from the widely held view that reviewer agreement is low: Based on overall agreement we consider the concordance among reviewers sufficient for the purposes of editorial decision making. We believe that various measures, such as positive and negative agreement or alternative Kappa values are superior to the application of Cohen's or Fleiss' Kappa in the analysis of nominal or ordinal level data regarding reviewer agreement. Also, reviewer recommendations seem to be a poor proxy for citations because, for example, manuscripts will be changed considerably during the revision process.

## Introduction

Peer review has become the cornerstone of scientific evaluation in medicine. Introduced already in the 18^th^ century by the journal *Philosophical Transactions* [1] it came into widespread use after World War II [2]. Although it has been criticized as hampering innovation [3] it now seems to be indispensable for quality based decision making in medical science: abstract acceptance at scientific meetings, grant allocation by private or state run research sponsors, promotion in academia, and manuscript acceptance or rejection at scientific journals – all evaluations rely on peer review. Today, apart from medical newspapers, society newsletters, and some industry sponsored for-free journals, all medical journals appear to use some sort of peer review in their evaluation process.

Medicine is a discipline largely built on journal articles, particularly in research. Also, much of continuing medical education of doctors rests on journal articles. Those articles and the journals where they appear differ in many aspects: for example, general medical versus specialty journals, English language versus regional language journals, or research papers versus review articles. Those differences will probably be reflected in different peer review processes and results. Against this background and given how essential and ubiquitous peer review has become, it is necessary to evaluate manuscript peer review at journals. Important questions pertain to the reliability of peer review, or to its validity, operationalized, for example, as its predictive value for scientific quality (measured as, e.g., citations).

Given the sheer number of medical journals – as of July 2012, NLM's database Medline alone indexed more than 5600 periodicals [4] – it is surprising that only relatively few have analyzed their peer review process and published the results (for a recent review of the research across academic disciplines and across reviewed material see [5]).

Several authors concluded that peer review is an unreliable process: For example, Kappa values as a measurement of inter-rater reliability have repeatedly been reported to fall between 0 and 0.4, indicating only poor to fair agreement among reviewers [5]–[9]. However, Cohen's kappa is not necessarily a reliable measurement of agreement and, under certain conditions, has been shown to be a flawed measurement of inter-rater reliability [10]–[12].

To our knowledge, only one study has measured the predictive value of reviewer recommendations for scientific citations in medicine: Opthof and co-workers [13] reported a significant but low correlation (0.14) between priority recommendation by a reviewer and the number of citations to a published article. Like many others, this study has been conducted at a specialty journal.

Accordingly, we analyzed the peer review process at our general medical journal focusing on the following questions: What is the distribution of reviewer recommendations? To what degree did the editors follow reviewer recommendations? What is the agreement among reviewers in evaluating manuscripts? Are reviewer recommendations associated with the number of future citations?

## Methods

This is a retrospective cohort study regarding reviewer assessments and citations of manuscripts submitted to Deutsches Ärzteblatt International. Deutsches Ärzteblatt International is a weekly, bilingual (German and English) general medical journal that publishes approximately 100 original and review articles per year. It is the official organ of the German Medical Association (Bundesärztekammer) and of the National Association of Statutory Health Insurance Physicians (Kassenärztliche Bundesvereinigung). As such, it is geared at the general medical public and not a specialty journal. A large share of its articles serves educational purposes. The (German) print version is distributed to all doctors in Germany, resulting in a circulation of more than 400.000 copies. In addition, all article full-texts are published (open access) in German and English via the webpage of the journal (www.aerzteblatt-international.de). In general, manuscripts are submitted in German and are reviewed by German speaking referees.

All manuscripts that underwent peer review that is, all original and review article manuscripts, submitted to Deutsches Ärzteblatt International between July 1, 2008 and December 31, 2009 were included in the present analysis. Not considered are editorials, letters to the editors, or congress reports. All manuscripts sent out for peer review were evaluated by at least two referees. Reviewers were selected, often in collaboration with the editorial board and following a literature search in medical databases, according to their area of expertise and to earlier experiences at the journal. They were asked to fill out a questionnaire including an overall recommendation whether the manuscript should be published. The four types of recommendations were: 1. Acceptance without revision, 2. Acceptance after minor revision, 3. Acceptance after major revision, 4. Rejection. Reviewers were not blinded with regard to the authors of a manuscript, and editors, in their decision-making, were aware of both the authors' and the reviewers' identity. Re-reviews (evaluations of manuscripts revised according to reviews by the same expert) are not included in this analysis.

For all manuscripts eventually published, a citation count was carried out using the Web of Science database. All citations until December 31, 2011 were counted.

### Statistical analysis

Manuscript submissions, acceptance and rejection rates, numbers of reviews, and their recommendations are presented as numbers, percentages (including 95%-confidence intervals), means, and standard deviations as appropriate.

For the purposes of the editorial office, reviewer recommendations 2 and 3 (accept after minor or major revision) boil down to the same suggestion: The authors should be invited to revise their manuscript. In addition, in our experience comments by reviewers that had proposed minor or major revision are not reliably different in length or specificity. Therefore, we merged recommendations 2 and 3 into a single recommendation of acceptance after revision: 1. Acceptance, 2. Acceptance after Revision, 3. Rejection. This rating was used for all further analyses including the calculation of mean recommendation scores. The reviewer recommendation score for a particular manuscript was calculated as the average of the recommendations of all referees.

### Statistical analysis of reviewer agreement

Several indicators of agreement are calculated to present different perspectives on the analysis of agreement measurement. A contingency table (3×3, using accept without revision, accept after minor/major revision, and reject as categories) is used to document recommendations of reviewer pairs. For those pairs, Spearman's correlation coefficient was calculated. Overall agreement of reviewer recommendations is presented as percentage and as positive and negative agreement rates for ‘accept with/without revision’ versus ‘reject’ – the latter calculated as described by Cicchetti and Feinstein [14] and generalized to >2 reviewers by Uebersax [15].

Furthermore, various Kappa values are presented. Fleiss' Kappa [16] was calculated in order to adjust for chance agreement and was preferred to Cohen's kappa on theoretical grounds: Unlike Cohen's Kappa, Fleiss' Kappa is valid for >2 reviewers per manuscript and for the case where (in general) different subsets of reviewers rate each manuscript. For comparison with the literature, Cohen's Kappa was also computed. Since Cohen's Kappa is limited to 2 reviewers per manuscript, all possible pairs of reviewer recommendations pertaining to a given manuscript were analyzed. For manuscripts undergoing peer review by more than two referees all possible reviewer pairs were constructed: For example, three reviews (A,B,C) resulted in three pairs of reviews (AB, AC, BC), and four reviews in six pairs. A secondary analysis was performed restricted to those manuscripts rated by just two reviewers.

However, Kappa values can be misleadingly low when the marginal totals are very different [10]–[12]. Therefore, an alternative chance-corrected statistic as proposed by Gwet [11], [12] was calculated. According to Landis and Koch [17], Kappa values denote the following levels of agreement: 0–0.2: poor, 0.21–0.4: fair, 0.41–0.6: moderate, 0.61–0.8: substantial, 0.81–1: almost perfect.

### Statistical analysis of reviewer recommendations and citations

The association of reviewer recommendations (mean of ratings of all reviewers per manuscript) and number of citations was analyzed using Spearman's correlation. Citations were counted between publication (2008 to 2011) and December 31, 2011. In order to adjust for different time spans during which a paper can be cited, year of publication was added as confounding variable in a partial correlation.

## Results

### Fate of submitted manuscripts

Between July 1, 2008 and December 31, 2009 a total of 343 original and review manuscripts were submitted to Deutsches Ärzteblatt. Of those, 137 (39.9%, [95%-CI: 34.9–45.2%]) were rejected after initial evaluation by the editors. The remaining 206 (60.1%, [54.8–65.2%]) were sent out for peer review, to 3 reviewers on average (mean: 2.7, range: 2–7). About one fourth (23.8%, [18.4–30.0%]) of the reviewed articles were rejected (n = 49), and the remaining 157 (76.2% of reviewed manuscripts, [70.0–81.7%]) were published. Of all manuscripts submitted to Deutsches Ärzteblatt (N = 343), eventually about half were published (n = 157 or 45.8%, [40.6–51.1%]).

### Number of reviews, recommendations and concordance with editorial decisions

The total number of reviews amounted to 554. In thirty-nine reviewer statements (7.0% [5.1–9.4%]) the recommendation was acceptance without revision and in approximately one fifth of reviews the referees opted for rejection (n = 107, 19.3% [16.2–22.8%]). In the vast majority acceptance after (minor of major) revision was recommended (n = 408, 73.6% [69.9–77.2%]; table 1).

**Table 1:** Reviewer statements.

Reviewer statement	N (%)
Accept without revision	39 (7.0%)
Accept after revision	408 (73.6%)
Reject	107 (19.3%)

N = 554 reviews.

When recommendations for acceptance and revision were aggregated, 127 out of 206 manuscripts (61.7% [54.9–68.1%]) sent out for review had a unanimous statement favoring acceptance. Almost all of those were eventually accepted (n = 118, 92.9% [87.4–96.5%]). The acceptance rate was lower for manuscripts with diverging reviewer statements, that is, acceptance or acceptance after revision versus rejection: 54.2% (39 out 72 [42.6–65.4%]). When all reviewers had advised rejection (n = 7) none of the manuscript were published (table 1). When we analyzed manuscripts that were evaluated by exactly two reviewers (n = 93) these figures did not considerably change (data not shown).

The probability for publication regarding a manuscript unanimously positively evaluated by the reviewers was 2.2 times higher than that of all other manuscripts, and probability for publication of manuscripts unanimously recommended for rejection was zero (table 2).

**Table 2:** Reviewer statements and editorial decisions.

Reviewer statements by manuscript	% (N)	Editorial decision: acceptance	Relative risk^1^ [95%-CI]
Unanimous: accept or accept after revision	61.7 (127)	92.9 (118)	1.88 [1.55–2.19]
Diverging: accept or accept after revision versus reject	35.0 (72)	54.2 (39)	0.62 [0.51–0.76]
Unanimous: reject	3.4 (7)	0	0.0 [0.0–0.56]

1 Probability (“risk”) of a manuscript in this group being published compared to the probability of a manuscript in both other groups.

### Concordance of reviews

#### Proportion of agreement

For all 206 papers evaluated in 554 reviews, 529 reviewer pairs were constructed (when more than two experts reviewed a manuscript more than one pair of reviewer statements could be analyzed). The overall rate of concordant pairs of statements was 60.9% (n = 322); acceptance without revision: 2; acceptance after minor or major revision: 289; rejection: 31 (table 3). When this analysis was repeated for the 93 papers evaluated by exactly two referees, the results were similar: 58 (62.4%) concurred: in 54 cases both experts opted for acceptance after revision and four manuscripts were unanimously recommended to be turned down.

**Table 3:** Agreement among reviewers regarding recommendations on the publication of evaluated manuscripts (N = all 529 possible reviewer pairs from 554 reviews on 206 manuscripts).

		Reviewer 2*			
		Accept	Accept after revision	Reject	
**Reviewer 1** *	**Accept**	2	36	2	40
	**Accept after revision**	35	289	62	386
	**Reject**	3	69	31	103
		40	394	95	529

Cohen's kappa for this 3×3 cross table: 0.059 [95%-CI: −0.016–0.134], Spearman's r: 0.17 (p<0.0001). When “accept” and “accept after revision” were condensed into one variable indicating acceptance Cohen's kappa for the resulting 2×2 cross table was 0.16 [95%-CI: 0.059–0.252], Spearman's r: 0.16 (p = 0.0002).

* Designation of reviewers as ‘Reviewer 1’ or ‘Reviewer 2’ was arbitrary.

When the options acceptance without revision and acceptance after revision were condensed so that the 3×3 table became a 2×2 table the results differed slightly: Overall agreement was 74.3% (n = 393/529). Again, the figure was similar when only the 93 papers were analyzed that were reviewed by exactly two referees: 75.2%. The proportion of positive agreement (concerning acceptance) was 0.84 (84%) and the proportion of negative agreement (concerning rejection) was 0.31 (31%).

#### Kappa values

Fleiss' kappa was 0.16, indicating poor agreement, and equal to Cohen's kappa: 0.16. The alternative kappa value (AC) by Gwet indicated substantial agreement: 0.63.

### Prediction of citations by reviewer statements

**Figure 1:**
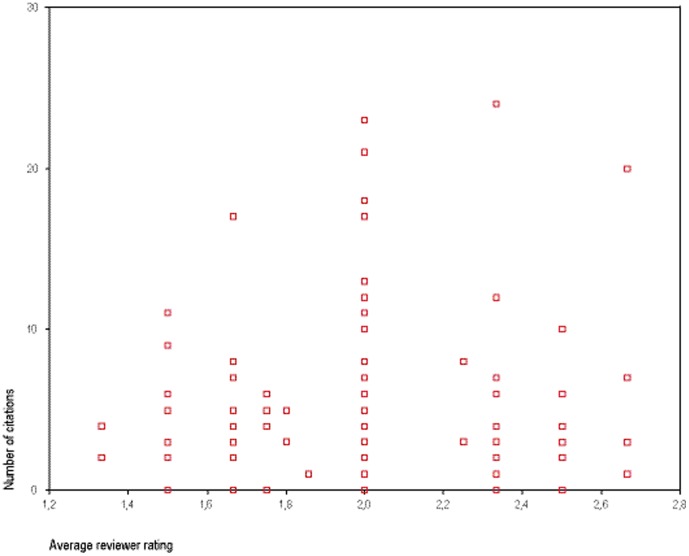
Scatterplot of average reviewer rating (x-axis) and number of citations (y-axis). N = 157 articles published in Deutsches Ärzteblatt International 2008–2011. X-axis: Reviewer recommendations: 1 denotes accept without revision, 2 denotes accept after minor/major revision, and 3 denotes reject. Spearman's r: −0.06; partial correlation, adjusted for year of publication: −0.03 (n.s.).

The 157 articles submitted between July 1, 2008 and December 31, 2009, and eventually accepted by Deutsches Ärzteblatt International were published between 2008 and 2011. Mean reviewer rating per article was 2.12 (SD: 0.35). On average, they were cited 5.1 times (SD: 4.9) between publication and December 31, 2011 (Figure 1). The correlation of average reviewer score for an article (lower scores indicate higher quality) and the number of cites to this article was −0.06 ( p = 0. 47). When, in a partial correlation, the association was adjusted for the year of publication the correlation dropped to −0.03 (p = 0.70).

## Discussion

This study yielded several relevant results: Firstly, at Deutsches Ärzteblatt International referees very rarely recommended acceptance of a manuscript in unchanged fashion. A substantial share of manuscript evaluations recommended rejection of the manuscript whereas the majority recommended revision and publication. Secondly, almost all unanimous recommendations (rejection or acceptance) were followed by the editors, and when reviewer statements differed (at least one reviewer opted for rejection and at least one for acceptance) about half were finally accepted. Thirdly, and this may be the key finding of our study, reviewers agreed substantially in their recommendations to accept (with or without revision; agreement rate 84%) but not in their recommendations to reject (agreement rate 31%). This agreement was not reflected by Fleiss' (or Cohen's) kappa (0.16) but by the alternative kappa statistic as proposed by Gwet (0.63). Fourthly, reviewer recommendations regarding an article did not predict how often it was cited.

### Distribution of reviewer recommendations

About one fifth (19.3%) of all reviewer statements at Deutsches Ärzteblatt International came with a recommendation to reject the manuscript under review. This is similar to the 17% published for the Croatian Medical Journal [7] and not far away from the 28% at the Journal of General Internal Medicine [8] – two other general medical periodicals. For two unnamed specialty journals in neuroscience, Rothwell and Martyn [6] reported higher rates of rejection recommendations (29 and 39%, respectively). Of note, the share of rejection recommendations among all reviewer statements is influenced by the editorial process: If all manuscripts are screened and pre-selected in the editorial office, as is the case at Deutsches Ärzteblatt International and other general medical journals, the proportion of articles rejected by the referees will likely be smaller than without such a pre-selection procedure.

Various reasons may prompt reviewers to opt for turning down a manuscript, e.g., poor scientific quality, the feeling that a manuscript is better suited to another type of journal (specialty journals versus general medical journals), or that the work under review is simply not good enough for a particular journal. For example, with 44% rejection was often proposed by the reviewers of The Lancet [7], possibly reflecting that referees grade manuscripts according to the journal that asked for a review.

### Concordance of editorial decisions and reviewer recommendations

During the observation period, four out of ten submissions to Deutsches Ärzteblatt International were rejected without review, often because the editors had deemed the material inappropriate for the journal's general readership. However, it is possible that reviewers are not aware of the fact that once they are asked to evaluate a manuscript the editors have already decided that it is in principle suited for their journal.

In general, the editors at Deutsches Ärzteblatt International followed reviewers' recommendations when they were unanimous for acceptance or rejection. Only in rare exceptions were papers rejected that the reviewers had unanimously considered publishable. Reasons included an evident divergence between a reviewer's recommendation and his or her specific comments or when authors did not comply with reasonable revision proposals.

### Agreement of reviewer recommendations

With regard to the recommendation to accept or revise a manuscript, agreement among reviewers was substantial. At Deutsches Ärzteblatt International one particularly important purpose of the review process is to guide the editors in their decision on whether to invite the authors to revise their manuscript or to reject. No manuscript that was eventually published was accepted without revision. Therefore, it is justified to condense all three recommendations –accept as is, revise, reject – into two: revise or reject. In three out of four pairs of manuscript evaluations this alternative was unanimous. More specifically, we found a high proportion of positive agreement (0.84) indicating that a decision to accept the manuscript (possibly conditional on revision) was largely unanimous. 84% of such decisions by one reviewer were in agreement with a given second reviewer. However, the low proportion of negative agreement (0.313) shows that the decision to reject was by no means unanimous, such decisions being supported by a given second reviewer in only 31% of cases.

For the practical purposes of an editorial office we consider this pattern of agreement satisfactory, particularly with regard to the acceptance of manuscripts.

The substantial agreement among reviewers, however, was not reflected in the conventional kappa statistics. Fleiss' and Cohen's kappa were low: 0.16 in both cases, respectively – values that are usually interpreted as poor agreement [17]. Of note, in this study and also in the studies presented in table 4, Fleiss' and Cohen's kappa are almost identical. This, however, is not necessarily the case.

**Table 4:** Various measurements of reviewer agreement in three studies involving manuscript review at medical journals.

Study	Journal (# of manuscripts)	Overall agreement	Positive agreement	Negative agreement	Cohen's Kappa	Fleiss' Kappa	Gwet's Kappa
Rothwell & Martyn 2000	Journal A (179)	57%	65%	45%	0.10	0.09	0.18
	Journal B (116)	71%	77%	60%	0.37	0.37	0.46
Yadollahie et al. 2004	Iranian J Med Sciences (28)	46%	44%	48%	−0.07	−0.07	0.12
Baethge et al. 2012	Dtsch Arztebl Int (206)	75%	84%	31%	0.16	0.15	0.63

Positive agreement denotes agreement regarding acceptance, negative agreement refers to agreement regarding rejection of a manuscript.

This situation, poor agreement as indicated by conventional Kappa statistics, is similar to the low kappa scores reported by Kravitz et al. [8] – with 55% agreement – and by Rothwell & Martyn [6] – with 57 and 71% agreement, respectively. Also, Bornmann and co-authors [5], in their essential systematic meta-analysis, calculated the mean kappa score of 26 investigations to be 0.17 (95%-CI: 0.13–0.21) and concluded that inter-rater reliability was low and that higher scores may be indicative of methodological flaws.

However, when certain recommendation categories are selected much more frequently than others, as in the present study, it may be problematic to rely on Fleiss' or Cohen's kappa with its specific calculation of chance agreement that is compared with actual agreement. Also, it has to be borne in mind that Cohen's Kappa will depend upon the arbitrary assignment of referees as reviewer 1 or 2.

Spearman's correlation coefficients for reviewer agreements were below 0.2, indicating poor agreement (table 3). This may be partly due to the small number of possible values (accept, accept after revision, reject) and the consequently large number of ties.

Gwet [11], [12] has argued that chance agreement should not exceed 0.5 in a 2×2 table. In fact, in a 2×2 table the pre-test chance agreement is 0.5. Gwet [11], [12] proposed an alternative kappa statistic that differs in the way chance agreement is calculated (table 5) and is particularly useful when inter-rater agreement is high. When we applied this alternative kappa statistic to our results and to the results of similar studies– provided enough data were available –higher scores resulted (table 4): The alternative kappa for our figures was 0.63, indicating substantial reliability. Interestingly, studies using intra-class correlation, a reliability measurement for continuous data similar to Cohen's kappa in considering chance agreement and in its interpretation, reported higher values on average (0.34, indicating fair reliability), as reported in the meta-analysis by Bornmann et al. [5]. For studies on reviewer agreement, we recommend to report the proportions of positive and negative agreement as described by Cicchetti and Feinstein [14] and to consider the alternative chance corrected kappa as proposed by Gwet [11], [12].

**Table 5:** Exemplary calculation of the alternative kappa statistic as proposed by Gwet (2002).

		Reviewer 1		
		Acceptance	Rejection	Total
Reviewer 2	Acceptance	A	B	B1 = A+B
	Rejection	C	D	B2 = C+D
	Total	A1 = A+C	A2 = B+D	N

Alternative chance agreement probability:

e(y) = 2P_1_(1-P_1_).

Approximate chance that a rater (A or B) classifies a subject into category “acceptance”.

P_1_ = [(A1+B1)/2]/N.

Alternative kappa statistic:

AC1 = [p-e(y)]/[1-e(y)].

Where p = (A+D)/N.

In sum, our findings challenge the view that journal peer review, in general, is unreliable.

### Prediction of citation by reviewer recommendations

Average reviewer recommendations and citations of published manuscripts were only weakly and insignificantly correlated. When the year of publication was taken into account in order to adjust for the different duration of time periods during which a paper could be cited the association became even weaker. This result is in line with the low correlation reported by Opthof and co-authors [13]: 0.14 for manuscripts evaluated by two reviewers. Bornmann and Daniel, however, in a study of the chemistry journal “Angewandte Chemie” [18], have shown that the number of citations (counted in Angewandte Chemie and in other journals) and the decision of Angewandte Chemie's editors to accept or reject a paper were substantially associated.

A low predictive value of reviewer recommendations for citations may indicate poor review quality and, therefore, low validity of the reviewing process. However, in part, the result may be explained by the fact that reviewers come to their conclusions at the beginning of an often time-consuming process of revising a manuscript. Therefore, accepted versions of manuscripts differ considerably from submitted versions. In addition, depending on their idea of evaluating a manuscript for Deutsches Ärzteblatt International, experts may consider an article of educational but not necessarily of scientific merit. Also, by definition, all manuscripts that were published reached high scores. It is, therefore, possible that variance of the reviewer recommendation was low precluding high correlations. In addition, the range of citations is restricted in articles published in Deutsches Ärzteblatt International because certain often cited types of articles (such as RCTs) are rarely published in our journal. In order to obtain a representative assessment of the association of reviewer scores and citations, publications in a broad set of journals have to be analyzed. Finally, although highly valuable as a measurement of journal evaluation, citations of single articles may not be appropriate for analyzing scientific quality [19], [20].

### Limitations

This study has several limitations: First of all, this is an analysis of the review process at one German general medical journal during a limited time span, and its results are certainly not representative for other journals. For example, it is conceivable that the manuscripts, the reviewers, the percentage of experts declining to review (at our journal: 16%) or submitting their evaluations (3%), or other factors typical for our journal differ from other general medical journals. Still, while the concordance among reviewers measured at our journal seems to be larger than in some (not all) other analyses, most results are similar to what has been published before.

Another shortcoming is that this is a study of a convenience sample of manuscripts and no sample size calculation was carried out prior to study start: Our N (206) is below the average N of 311 that Bornmann et al. reported in their meta-analysis [5]. While a higher N would be desirable 206 manuscripts and 554 reviewer statements seem enough to yield a correct order of magnitude for most calculations. As an example, the confidence interval of the proportion of reviewer recommendations for revision seems sufficiently narrow: 69.9%–77.2%. And yet, it has to be borne in mind that all figures reported in this paper are estimates.

The citation analysis in this study is restricted to Web of Science and did not use other databases such as Scopus or Google scholar. However, while Google scholar retrieves more citations the citation accuracy is lower [21]. The difference between Web of Science and Scopus in the number of manuscripts retrieved is smaller but, because of its relationship to the impact factor, it is Web of Science that, for the time being sets the standards in scholarly publishing. Therefore, it was chosen as the basis of this analysis. Finally, the present analysis is restricted to the recommendations by the referees regarding publication. Other important purposes and aspects of the review process could not be covered: For example, it would be important to analyze the specific comments by the reviewers, either to the editors or to the authors. Those comments sometimes differ from the recommendations and may convey more subtle messages regarding, for example, the constructive improvement of manuscripts but also the possible inhibition of innovation or matters of power and discipline territoriality. Such an analysis would have to use qualitative methods.

## Conclusions

In an analysis of the review process at a general medical journal from Germany we found that reviewers more often recommended revision than rejection or straight acceptance. Most importantly, reviewers agreed substantially among themselves concerning acceptance but not concerning rejection; editorial decisions concurred substantially with unanimous reviewer recommendations; but recommendations and number of citations were not associated. Based on a comparison of the commonly used Cohen's (or Fleiss') Kappa statistic for measuring inter-rater reliability with an alternative Kappa statistic, we argue for a reconsideration of the widely held view that journal peer review is a process of poor reliability. Provided that our results hold up, implications of this study include the application of different agreement measurements (preferably as proposed by Cicchetti and Feinstein or by Gwet rather than applying Cohen's Kappa), and the knowledge that, in medicine, reviewer comments will not tell editors and authors much about future citations. Also, at least with regard to reviewer agreement, the peer review system seems to work well.

Many of the most important questions regarding peer review, however, such as whether peer review leads to streamlining of science and whether it hampers innovation, or to what degree peer review improves manuscripts, remain open, and qualitative research will probably be needed in the search of answers [22].
